# Simulation of Platelet, Thrombus and Erythrocyte Hydrodynamic Interactions in a 3D Arteriole with *In Vivo* Comparison

**DOI:** 10.1371/journal.pone.0076949

**Published:** 2013-10-02

**Authors:** Weiwei Wang, Thomas G. Diacovo, Jianchun Chen, Jonathan B. Freund, Michael R. King

**Affiliations:** 1 Department of Biomedical Engineering, Cornell University, Ithaca, New York, United States of America; 2 Department of Pediatrics and Pathology and Cell Biology, Columbia University Medical Center, New York, New York, United States of America; 3 Departments of Mechanical Science & Engineering and Aerospace Engineering, University of Illinois at Urbana-Champaign, Urbana, Illinois, United States of America; University of Leuven, Belgium

## Abstract

Cylindrical blood vessels, ellipsoid platelets and biconcave-shaped deformable erythrocytes (RBCs) are important participants in hemostasis and thrombosis. However, due to the challenge of combining these components in simulation tools, few simulation studies have included all of them in realistic three-dimensional models. In the present study, we apply a recently developed simulation model to incorporate these components and analyze the flow in a thrombotic tubular arteriole, particularly the detailed hydrodynamic interactions between the thrombus shape, RBCs and platelets. It was found that at certain azimuth positions, the velocity drops in the proximity of both the upstream and downstream edge of the thrombus, which is accompanied by a rapid velocity increase in the narrowed region. The RBCs alter the flow profiles significantly from the typical low Reynolds (Re) number flow, and also enhance the deposition of free flowing platelets onto the thrombus. By evaluating the platelet-thrombus interaction and platelet-RBC interaction together, several mechanisms of platelet deposition augmentation are identified. With *in vivo* data comparison, our model illustrates the potential of future thrombosis studies that incorporate detailed receptor-ligand adhesion modules.

## Introduction

Thrombosis, the pathological process of the hemostatic system to form unwanted blood clots, can impair blood flow to vital organs and ultimately result in stroke or myocardial infarction [1]. Although widely studied, the complicated ensembles of the hemostatic system, together with measurement limitations, motivate the development of computer simulations of the physiological processes of hemostasis and thrombosis. Current hemostatic simulation models consist of one or more of the following components: hydrodynamics, coagulation cascade, platelet activation, fibrin network, cell mechanics or receptor-ligand adhesion [2]. Some of these modules utilize a “systems biology” approach where the physical elements are described by concentration fields with advection and diffusion in the fluid (plasma) [3]. Others take the mechanics of those elements into consideration, such as shape, density and deformability [2,3].

The physical contact between flowing platelets and the injured vessel surface (including the developing thrombus) is a key event in the progress of thrombosis. Various simulation studies have been performed in an attempt to represent this hydrodynamic interaction. For instance, Mody and King applied the CDL-BIEM method to analyze the near-wall motion of a platelet in 3D and discovered three distinct regimes of platelet-wall hydrodynamic interactions [4]. Later, these authors simulated the platelet-platelet interaction near a wall and demonstrated the importance of the unique spheroid shape to the physiological function of the platelet [5]. Wang et al. used an immobilized platelet to address the early initiation of a micro-thrombus and studied the hydrodynamic interaction between an immobilized and flowing platelet [6]. Other platelet-vessel wall interaction models combine hydrodynamics with cell-wall adhesion. For example, Fogelson used an immersed boundary method to study the platelet-wall interaction with an adhesion module in 2D [7]; Mori et al. utilized Stokesian Dynamics to simulate platelet-wall interactions and thrombus development in 2D [8]. One of the first platelet-wall interaction models in 3D was presented by Pivkin et al., where the authors analyzed different thrombus growth patterns under different platelet activation delay times [9].

Interestingly, the platelet and vessel wall are not the only determinants in thrombus formation. Platelet margination, an elevated distribution of platelets towards the peripheral region of the vessel lumen, was discovered decades ago [10] and is believed to be mainly caused by the significant volume fraction of RBCs in whole blood. Simulation studies revealed that the high deformability of RBCs as well as the finite size of platelets compared to RBCs may be two reasons for such margination effects [11,12].

Experimental data comparison serves as an important process to evaluate simulation models. In recent years, due to the fact that more detailed models have been established along with more precisely controlled and measured experimental systems, such comparison is shifting from qualitative to quantitative. Flow chamber assays are a good source of *in vitro* data and examples have been shown to verify human platelet-wall interaction models [13]. Mouse models are typically used for generating *in vivo* data and are preferred by clinical investigators. However, they are limited by the fact that all hemostatic components are derived from mice [14]. A biological platform has been developed that specifically addresses this issue, by enabling the *in vivo* assessment of human platelet mediated thrombus formation [15]. This was accomplished by genetically modifying the A1 domain of murine VWF (VWF-A1) so that it interacts with human but not mouse GPIbα. Consequently, animals have a bleeding phenotype that can be corrected by the administration of human platelets. This humanized mouse model serves the purpose of *in vivo* comparison to our simulation model as we were able to monitor in real-time the dynamics of human platelet interactions with laser-injured arterioles.

In the present study, we utilized a high-efficiency 3D boundary integral method which evaluates the surface integrals using an *O*(*N logN*) particle-mesh Ewald (PME) approach [16] to simulate hydrodynamic interactions between a thrombus, flowing platelets, and surrounding deformable red blood cells (RBCs) with elastic membranes. The plasma flow at different locations inside the cylindrical vessel wall with an existing thrombus of various shapes was explored; the hydrodynamic effect of various degrees of stenosis was examined and compared to *in vivo* data; and the correlation between RBC-platelet interaction and platelet-vessel wall interaction was also assessed.

## Materials and Methods

All animal experiments were carried out in accordance with institutional IACUC committees of Cornell University and Columbia University, following protocols approved by both institutions’ IACUC.

### Hydrodynamic Calculation

The hydrodynamic calculation uses a previously reported fast boundary-integral algorithm for numerically computing the flow solution [16]. In this approach, the Green’s functions of the flow equation, which is linear in the viscous limit appropriate for the low Reynolds numbers of the microcirculation, are built into integral expressions, which relate the velocity to the forces exerted on the fluid by the elastic cells and the vessel walls. Note that while the fluid mechanics are linear, the combined cell-fluid system has significant geometric nonlinearity, so the collective behavior of the cellular is expected to deviate significantly from what would be predicted overall for a linear Stokes flow. The basic analytic formulation has been available for some time and is summarized clearly by Pozrikidis [17]. To discretize this formulation, the vessel walls and cell surfaces are represented by discrete elements. With such a discretization, numerical evaluation of the integral expressions is tantamount to forming a dense linear system to solve. It is dense because of the long-range interactions embodied in the Green’s function: all points on all surfaces induce velocities at all other points in the domain. This is prohibitively expensive to evaluate directly for systems of the size targeted by the presented study, scaling at least as O(N^2^), where *N* is the number of discrete points. However, an Ewald splitting of the Green’s function facilitates an approximate, though accurate, discretization that has a much better O(*N* log *N*) complexity [16,18]. This employs a periodic form of the Green’s function kernels, which is advantageous in the present cases since the target simulation is streamwise periodic, and not restrictive in the other directions since the presence of the vessel wall breaks any periodic influence.

### Cell Mechanics

The cell membranes were each discretized with a structured mesh interpolated by spherical harmonic basis functions, which were used to evaluate the elastic stresses. The membranes were mechanically modeled as elastic shells [19], which strongly resists areal dilatation and has a relatively weak resistance to in-plane shear. A bending resistance was also employed. The elastic parameters are those of Pozrikidis [20], which have been used with this simulation tool to reproduce the effective viscosity versus diameter for flow in round tubes of diameters 5µm up to about 30µm [16]. While this or any constitutive model will be unable to reproduce all conceivable red blood cell behaviors, this one is widely used and has been validated for flow in the present regime.

The spherical harmonic basis functions provide excellent resolution and convergence [16]. Since they facilitate error-free interpolation to finer meshes, they also enable a de-aliasing procedure for stabilization without the addition of numerical dissipation or filtering, which will in general degrade solution fidelity. The resulting scheme is both accurate and robust, so we can choose the resolution needed to achieve necessary accuracy for meeting objectives with relatively little regard to maintaining numerical stability. The resolutions used in the present simulations are consistent with those used previously and in other studies [21,22].

The platelets were modeled as rigid ellipsoids, with shape and size matching that used in past Platelet Adhesion Dynamics (PAD) studies [23,24]. This is appropriate given the relative stiffness of platelets, but introduces an additional challenge to the numerical solution since it renders the basic formulation singular [17]. We resolve this, as we have in studies of transport of magnetic nanoparticles [22], using the methods discussed by Kim & Karrilla [25] and Pozrikidis [17], by which the solid-body modes are projected out of the system and solved separately before being reincorporated to evolve the platelet positions. Our implementation was validated against the results of Hashimoto [26] for drag on an array of spheres (data not shown).

The vessel walls were represented by linear triangular boundary elements.

### Simulation Domain

We consider a streamwise periodic cylindrical vessel with diameter 15µm and length 45µm. In a vessel of this size, the Fahaeus effect [27,28] can be quite significant. The hematocrit value (volume fraction of RBCs in the blood, abbreviated as Hct) can drop from 40~45% in arteries of normal adults to 10~20% in small capillaries. A Hct of 10% is examined here, which corresponds to eight RBCs positioned inside the 45µm arteriole section. The platelet count in a healthy individual is between 150,000 and 450,000 per µL [29] which is equivalent to 1~3 platelets in the simulation volume. Computationally, we consider a single platelet, corresponding to a concentration of approximately 150,000 cells per µL (with consideration of volume exclusion of the thrombus). Larger vascular sizes, higher Hct values and platelet count may be employed in future studies at a higher computational cost. The initial configuration of the RBCs and platelet were predetermined manually, and simulations were run until they randomized and reached a statistically stationary state (data not shown). The platelet was placed at the peripheral region because of the well-known platelet margination effect [10,11,30]. The mean flow velocity was set at a velocity of 1128 µm/s, which is within the physiological range in arterioles of this size [31]. The resulting Reynolds number is about 5.65×10^-3^, which is well within the range of Stokes flow calculations.

A 3D bump was centered at the midpoint of the vessel to represent an existing thrombus and partial stenosis. Its specific shape is

$$y=H-\frac{w}{1.9^{\left(\frac{{\left(z-22.5\right)}^{2}}{a}+\frac{x^{2}}{b}\right)}}$$

which is based on *in* *vivo* fluorescent microscopic images from the mouse experiments of this study. The *x*-*z* section of the thrombus is an ellipse and the *x*-*y* section of the thrombus appears Gaussian in shape. By varying the parameters *a*, *b*, *w* and *H* one can obtain different thrombus shapes and percentages of stenosis (calculated as the *x*-*y* cross sectional area blockage).

### In Vivo Thrombus Formation

The VWF ^R1326H^ mouse strain was used as described in previous studies [15]. The administration of anesthesia, fluorescent labeling, and administration of human platelets and surgical preparation of the cremaster muscle in mice have been previous described [32,33]. Injury to the vessel wall of arterioles (~40–65 µm diameter) was performed using a pulsed nitrogen dye laser (440 nm, Photonic Instruments) applied through a 20× water-immersion Olympus objective (LUMPlanFl, 0.5 NA) of a Zeiss Axiotech vario microscope.

Human platelet–vessel wall interactions were visualized by ﬂuorescence microscopy using a system equipped with a Yokogawa CSU-22 spinning disk confocal scanner, iXON EM camera, and 488-nm and 561-nm laser lines to detect BCECF-labeled platelets (Revolution XD, Andor Technology). The extent of thrombus formation was assessed immediately after laser-induced injuries for a total observation period of 10 min. Images were captured at an average of 66.7 fps with 10 ms exposure time at an appropriate *x*-*z* plane. Data from three mice yielded ~4000 frames of micrograph images to analyze in this study.

### Image Processing and Analysis

The *in vivo* image stacks were processed using ImageJ. All the data visualization and analysis was carried out using Microsoft Excel and in-house developed Matlab codes.

## Results

First, we studied the blood flow profile in thrombotic vessels and the disturbed flow around the existing thrombus. The near-wall velocity field indicates potential platelet-wall interactions. A stability analysis on the hydrodynamics caused by RBCs was also performed. The stenotic flow velocity was compared to *in vivo* data and good agreement was found. Platelet trajectories when passing by the thrombus with and without RBCs were then examined. The platelet-thrombus and platelet-RBC interaction patterns were studied to evaluate the significance of RBC-enhanced platelet-thrombus interactions.

### Simulation of Flow Disturbance Caused By A Thrombus

**Figure 1:**
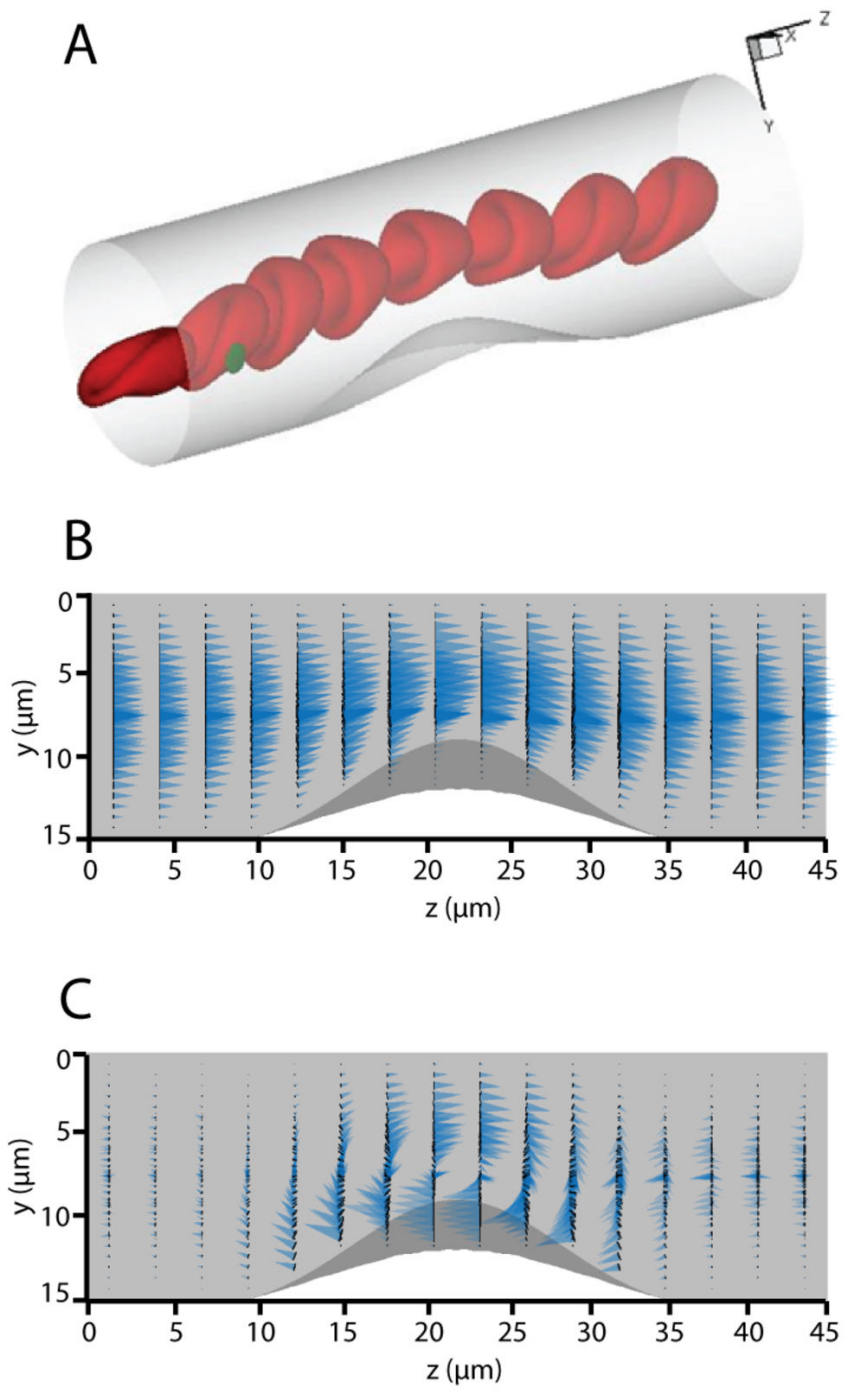
The 3D visualization of the simulation model and velocity profile inside the vessel lumen. (A) A representative sketch with one periodic boundary unit displayed. The red particles show the natural shape of biconcave-shaped RBCs under tubular flow. The green cell represents a platelet and the semi-transparent bump on the vessel wall represents the thrombus. (B) The velocity profile inside the vessel lumen is shown. Each cone represents the velocity vector at that specific location. (C) Deviation velocity field surrounding the thrombus.

The flow profile in the vessel section unit is displayed in Figure 1. The biconcave-shaped RBCs deform into cap-like structures in the cylindrical vessel lumen, in agreement with in vitro observations [34]. The velocity field is shown in Figure 1B. Along both ends of the *z*-axis, the velocity field shows an approximately parabolic shape with *z*-axis symmetry. The effect of the thrombus is localized, showing that the streamwise periodic length of the vessels is sufficiently large to avoid any significant artifacts associated with the non-*z*-axis symmetric periodic boundary conditions. When the flow reaches the thrombus (*z* = 15 to 30 µm), the entire flow is displaced in the *y*-direction. Figure 1C shows the disturbance of the flow caused by the thrombus. It was generated by subtracting the non-disturbed flow (flow inside a smooth cylindrical vessel without a thrombus) from the flow shown in Figure 1B. Note that the length scale of the blue cones is expanded in Figure 1C compared to Figure 1B to display the velocity disturbance clearly. It can be readily seen that the velocity disturbance at *z* = 5 µm is very small, verifying that the disturbance of the thrombus one period upstream has little impact on the target section. Indeed, it is known from the Green’s function of Stokes flow that the velocity disturbance from a point force decays at a rate of 1/*r*. The existence of the thrombus slows down the overall flow, dragging the near-thrombus flow by the no-slip boundary condition while accelerating the flow at the back side (locations with smaller *y* coordinate, as opposed to the thrombus side), showing how the fluid accelerates when passing through a narrowing blood vessel. It is worth noting that due to the existence of RBCs, the velocity profile does not display a smooth outline. In the model, the higher viscosity of the RBC cytosol as well as the elastic cell membrane [35] cause the velocity field to deviate from an smooth curve.

### Simulation of Flow Profile with Various Thrombus Shapes

**Figure 2:**
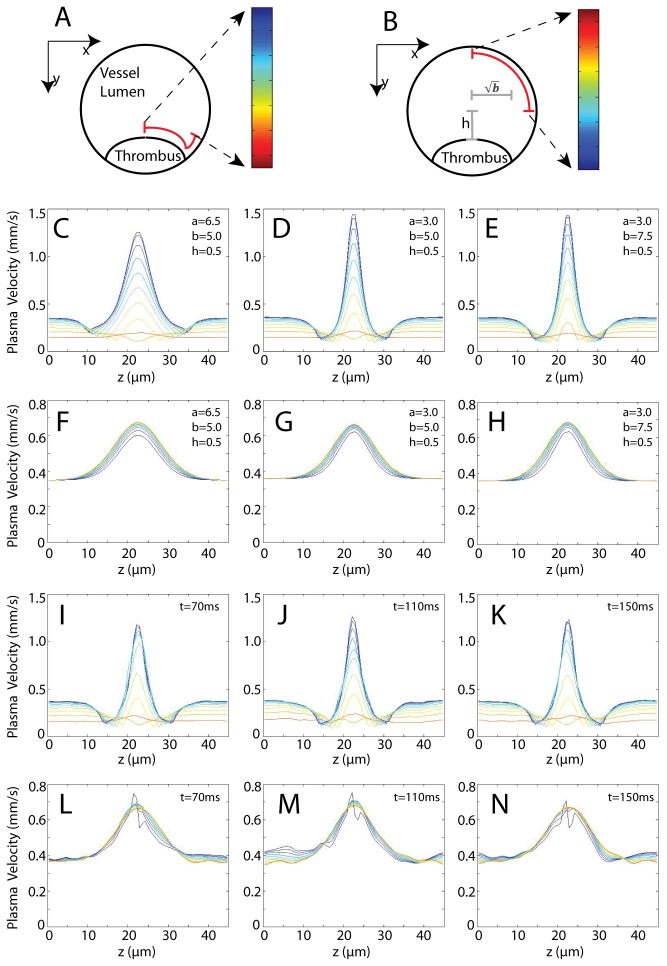
Near-wall velocity profiles. (A) Analysis geometry for C–E and I–K. The color bar identifies the series of near-thrombus velocity profiles in C–E and I–K along the curve (*y*-distance 0.5 µm from the wall). (B) Analysis geometry for F–H and L–N, with the color as in (A) but with 0.5 µm distance from the wall. (C–H) The velocity profiles with three different thrombus shapes are shown (Hct=0). (I–N) The velocity profiles at the same conditions as D and G, with Hct=10% are shown. The thrombus geometry parameters *a*, *b*, *h* = *H*-*w* are defined in Eq. 25 and the resulted degree of stenosis are 36%, 36% and 39% separately.

The near-wall velocity is shown in Figure 2. The velocity profile at the near-thrombus region (Figure 2C-E, I-K) and on the opposite side of the vessel (Figure 2F-H, L-N) was analyzed for different thrombus shapes. Since all of the velocities in Figure 2 are evaluated at the same distance from the vessel wall, this provides an estimate of the wall shear stress $\tau \approx \mu \frac{v}{\Delta y}$ (with Δ*y* being the same in all cases). As a result, the conclusions drawn for flow velocity in this section can be applied to shear stress as well. It was found that, at most of the locations along the central line of the thrombus, the near-wall velocity exhibited a drop directly before reaching and after passing the thrombus (Figure 2C-E, I-K), which matches previous observations [36]. Such stagnation zones are not found at the back side of the vessel, where the velocity changes at *z* = 22.5 µm are also smaller by three-fold (Figure 2F-H, L-N). It is interesting to note that not all azimuthal positions (as one color indicate one azimuth in Figure 2) experience a velocity increase when approaching the thrombus. At specific azimuths (Figure 2C, D, I-K orange line), the velocity in fact decreases while approaching the thrombus. This is most likely because at the locations where the thrombus surface and cylindrical vessel wall surface intersect and form a near 90° angle, the no-slip boundary conditions (imposed by the thrombus surface as well as the vessel surface) strongly limit the flow velocity in these valley regions. For different thrombus shapes, there is no significant change in the character of the flow (Figure 2C-H). Extension of the thrombus along the *z*-axis causes a similar expansion pattern in the velocity profile (Figure 2C, F), while extension of the thrombus along the *x*-axis reduces the “valley” region discussed above (Figure 2E). Without cells in the system, the near wall velocity shows surface symmetry at the surface z = 22.5 µm (Figure 2C-E). Such symmetry is not present for cellular blood flow (Figure 2I-N). It was also found that the peak near-wall velocity decreases by about 15% when Hct=10% (Figure 2D, I-K), suggesting that the existence of RBCs caused blood rheology change (e.g. increased viscosity). In the presence of RBCs and platelets, velocity fluctuations as a function of time are observed (Figure 2I-N). These fluctuations can be as large as 10% of the average. The velocities near the center of the thrombus exhibit a similar “zigzag” change (Figure 2L-N) at different time points. This may be due to the similar configuration of the surface boundary conditions when cap-shaped RBCs pass by the thrombus, with the caps oriented downstream. Taken together, these data indicate that blood cells, particularly deformable RBCs, may not be neglected when constructing thrombosis models.

### Simulation of Flow Velocity with Various Degrees of Stenosis and its Comparison to Published Patient Data

**Figure 3:**
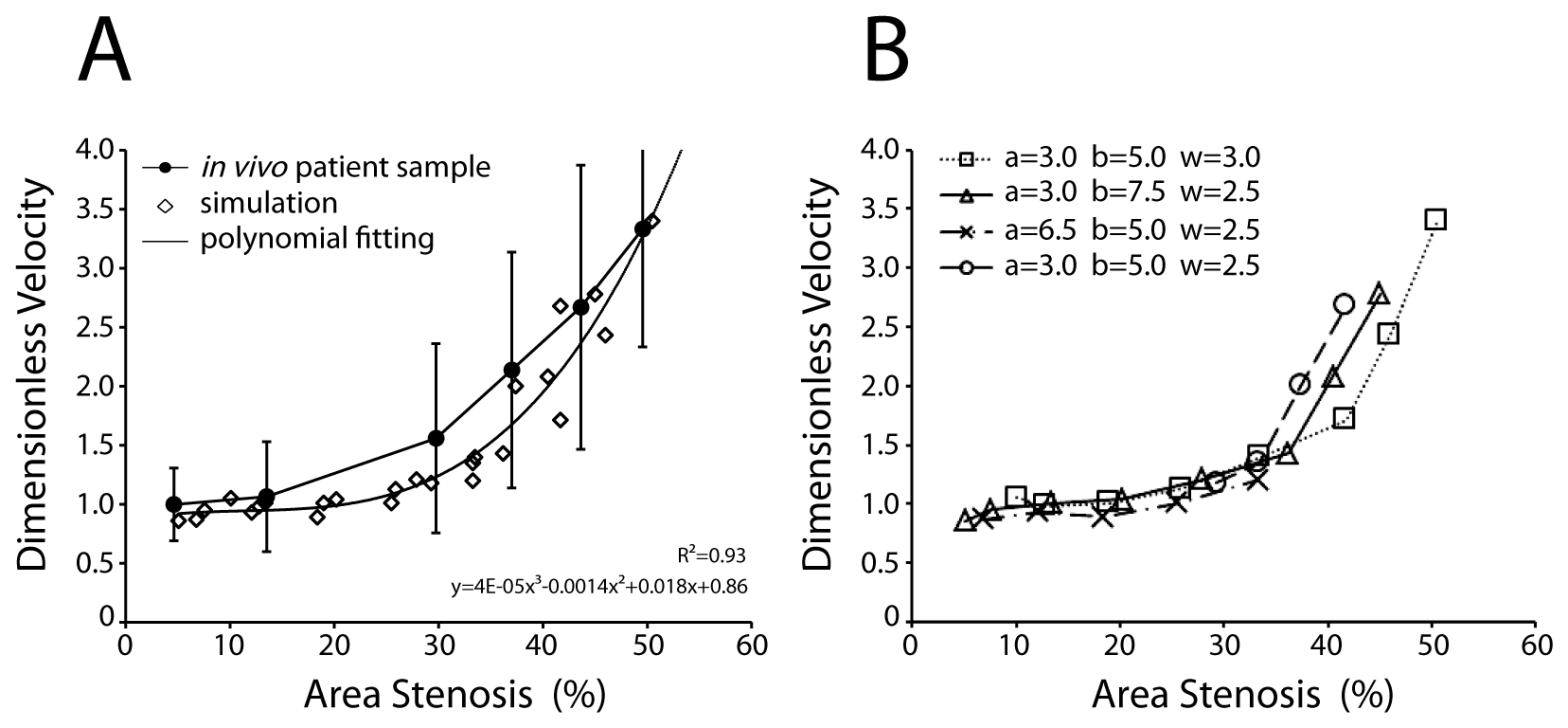
Peak blood velocity for a range of simulation settings and their comparison to *in vivo* data. (A) The dimensionless peak blood velocities (DPV) from four different thrombus settings were evaluated. Note: for each thrombus setting, the parameters *a*, *b* and *w* are fixed, but *H* was varied (Eq. 25) which resulted in different degrees of area stenosis. The percentage area stenosis was evaluated by the percent area blockage of the thrombus at *z* = 22.5 µm. (B) All the data points in (A) are plotted, fitted by polynomial fitting and compared to the *in* *vivo* carotid artery stenosis-blood velocity data [37]. (The *in* *vivo* data was non-dimensionalized with the *in* *vivo* average non-stenotic blood velocity of 75 cm/s.).

To confirm the hydrodynamics of our model, the dimensionless peak velocity (DPV) based on different degrees of area stenosis was calculated and compared to previously published patient data (Figure 3). The DPV is calculated by dividing the maximum *z*-axis velocity in the vessel by the maximum non-stenotic velocity. Several factors suggest the use of the DPV metric: (i), the consensus clinical indicator of stenotic blood velocity is the peak systolic velocity (PSV) [37], where the “peak” indicates the highest velocity which is usually found at the narrowest stenotic region (*z* = 22.5 µm in our cases, based on the results in Figure 2); (ii), non-dimensionalization enables direct comparisons. We found a super-linear relationship between DPV and the percentage area stenosis for all four thrombus shape settings (Figure 3A). The larger the percentage area stenosis, the greater the impact of thrombus shape on DPV, while for less-severe stenosis (<35%), the DPV do not vary much among different thrombus shapes (Figure 3A). Our simulation results agree with patient sample data where the blood flow velocity shows a super-linear relationship between DPV and percentage area stenosis (Figure 3B).

### Simulation of Platelet Trajectories and its Comparison to New Mouse Data

**Figure 4:**
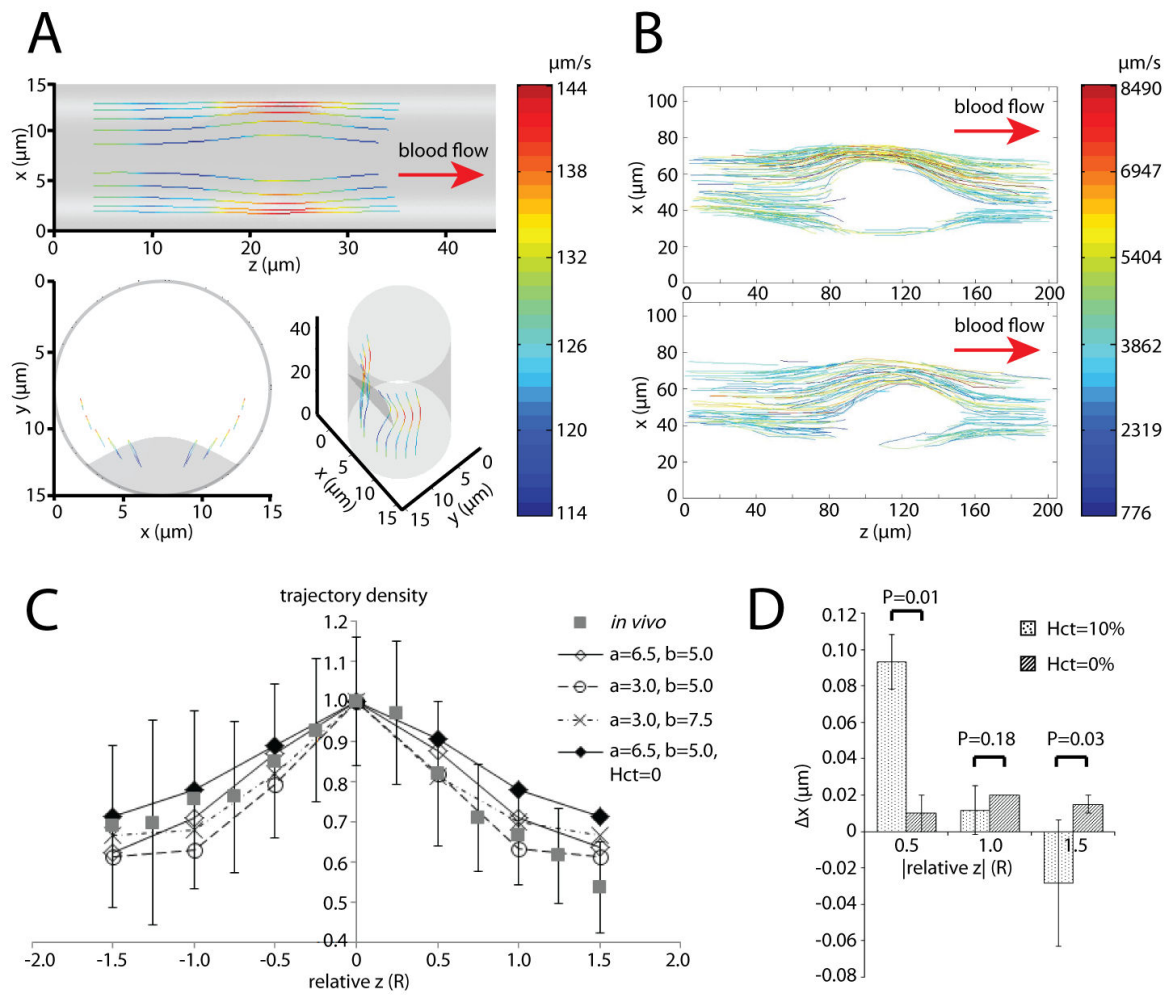
Platelet trajectories generated in simulations and compared with *in vivo* data. (A) A representative platelet trajectory collection generated by a series of simulations under a specific thrombus shape setting (*a* = 7.5, *b* = 6, *h* = 1.2, degree of stenosis = 23%) as viewed from three camera angles, with color indicating the local platelet velocity. (B) Two representative *in* *vivo* platelet trajectory collections are shown and color coded to represent the local platelet velocity. (C) Quantitative comparison between simulation and *in* *vivo* data of how platelet trajectories converge when passing by a thrombus and then diverge when leaving the thrombus. (D) Quantification of the *x*-coordinates difference of the platelet positions along a trajectory the same distance upstream and downstream the thrombus center (the absolute value of relative z coordinate) from the same series of simulations in (C). Two-sample unpaired t-test was performed with P-values given.

Platelet trajectories both in simulations and the *in vivo* mouse thrombus model (Figure 4A, B) were analyzed. *In vivo* platelet trajectory plot (Figure 4B) was reproduced by manually discretizing platelet trajectories over 500 frames. One way to quantitatively evaluate the hydrodynamic impact of the thrombus on platelet motion is to examine how platelet trajectories converge at the stenosis relative to the thrombus shape. We ran a series of simulations with platelets released at locations *y* = 0.5(22.5 -*h*) and the *x*-coordinates varied for each thrombus setting. Considering the limitations of fluorescent microscopy regarding the depth of field, simulations resulting in platelet trajectories fluctuating more than 2 µm in the *y*-direction were excluded. The trajectory densities were recorded according to their relative *z*-axis positions (Figure 4C), where the relative *z* is quantified by the *z*-axis position minus 22.5 µm and then scaled by the length of the major axis of the 3D thrombus projection (the projection is an ellipse as described) on the *x-z* plane. The trajectory density at each *z* = *Z*’ was calculated as: (i), projected all the trajectories onto the *x-z* plane; (ii), scaling of the total number of trajectories passing by the line *z* = *Z*’ by their largest *x*-coordinates separation (max(*x*)-min(*x*)); (iii), renormalization with the highest density. It is clearly seen that the extensions in both *z* and *x* direction (*a* = 3, *b* = 5) of the thrombus cause the platelet trajectories to converge and diverge at closer distances to the thrombus. It was also determined that the existence of RBCs drive platelets laterally thus further condensing the platelet trajectory when passing by the thrombus, compared to the less pronounced convergence and divergence in simulations without RBCs (Figure 4C). The reversibility of platelet trajectories, one of the key characteristics of Stokes flow, was checked. It was found that when RBCs are present, there is up to a 10% difference in the *x*-coordinates for the platelet positioned at the same distance upstream and downstream of the thrombus center along its trajectory (Figure 4D). On the other hand, there is only ~1% difference in the absence of RBCs which is mostly likely the result of numerical error. In other words, platelet trajectories do not reconverge downstream of the thrombus as rapidly as they diverge before reaching the thrombus. Two-sample unpaired t-tests indicate that this difference is significant (P <0.05) for most of the conditions (Figure 4D).

### Simulation Shows Direct Evidence That RBCs Enhance Platelet Deposition

**Figure 5:**
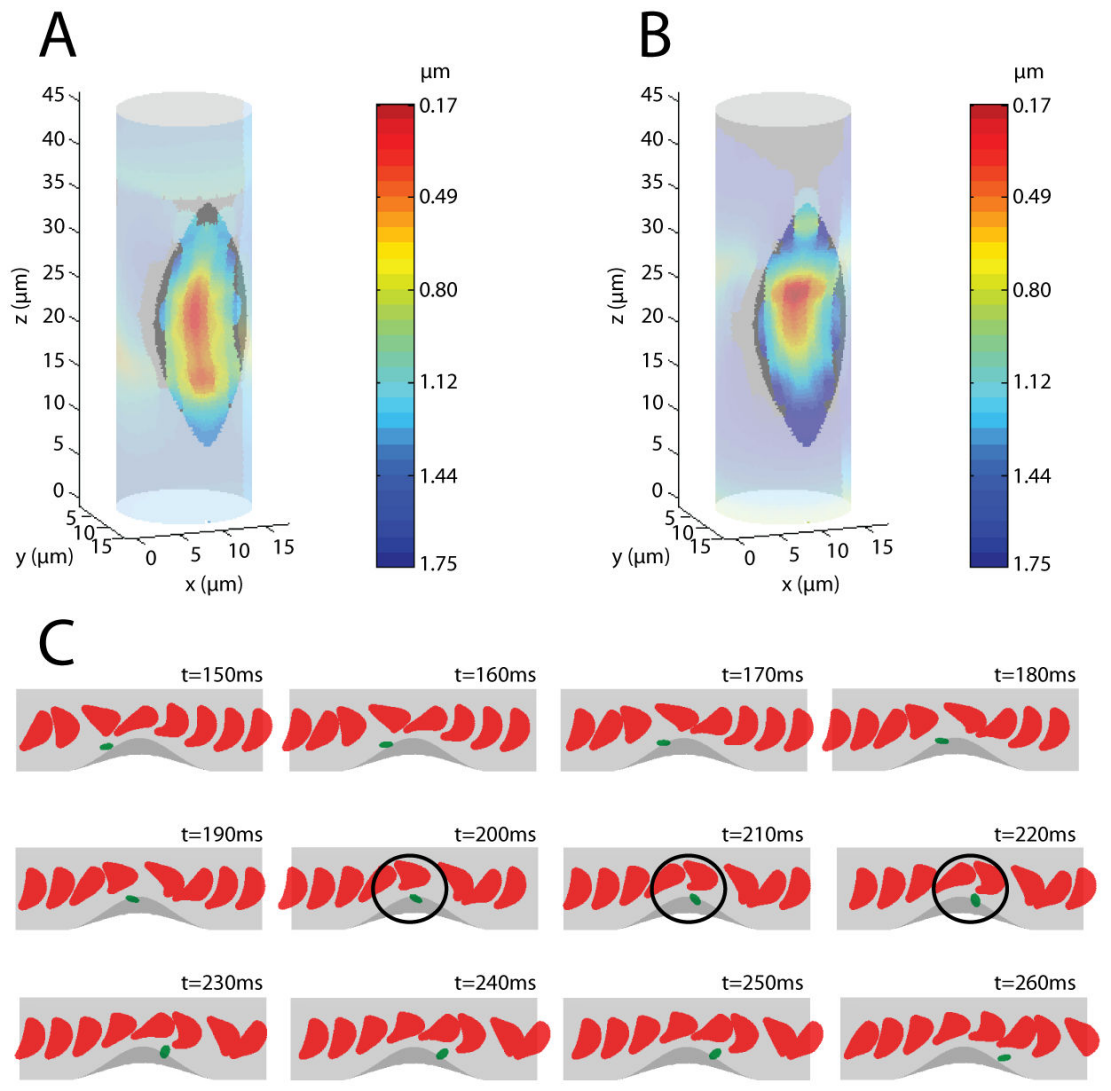
The platelet deposition potential is enhanced by hydrodynamic interactions with RBCs (thrombus settings: *a* = 7.5, *b* = 6, *h* = 1.2, degree of stenosis = 23%). (A) The platelet deposition potential map generated by a series of simulations with Hct = 10%. The color on the vessel wall surface indicates the distance between a flowing platelet and the vessel wall. (B) The platelet deposition potential map with the same settings as in (A) except Hct = 0. (C) Sequential frames of a representative simulation. The black circles demark direct evidence of RBCs-enhanced platelet-thrombus interaction.

To determine how RBCs affect platelet-thrombus interactions, the platelet deposition potential map (Figure 5A, B) generated by a series of simulation experiments with platelets starting at different peripheral positions inside the vessel was plotted. The platelet deposition potential is defined here as the shortest separation distance between the following platelet and the thrombus surface, with smaller distances corresponding to stronger potentials. A global enhancement of the platelet deposition potential on the entire thrombus surface is readily seen. The closest distance is about 0.17 µm, within the maximum reactive distance (~260nm) based on the approximation of the length of the integrin α_IIb_β_3_-vWF-α _IIb_β_3_ trimolecular bond [38,39]. The direct observation of RBC-enhanced platelet-thrombus interaction is depicted in Figure 5C. When the platelet follows the motion of a modified Jeffery orbit near the vessel wall, it rotates along the *x*-axis and transitions from a parallel orientation to a perpendicular orientation, then back to the parrallel orientation again [4]. During the perpendicular orientation, the platelet extends its reach along the *y*-axis and occupies the excluded volume generated by the RBCs. As a result, the platelet is pushed towards the thrombus thus enhancing its deposition potential (Figure 5C).

### Simulation of Platelets and Their Interaction with RBCs and Vessel Wall

**Figure 6:**
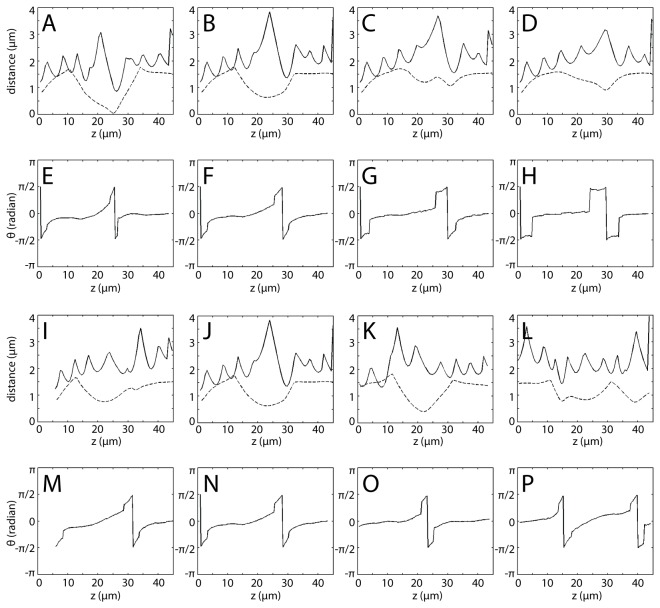
The hydrodynamic interaction between platelets, thrombus and RBCs and the configuration of the flowing platelet is shown. (A) -(D), (I)-(L) Solid lines display the shortest distance between the platelet and RBCs during platelet advection. Dashed lines display the shortest distance between the platelet and vessel wall surface. (E) -(H), (M)-(P) The rotational angle of the platelet about the *x*-axis is shown during platelet advection. (A) (E), (B) (F), (C) (G), (D) (H) are pairwise data generated from four simulations with platelets starting at *z* = 0 µm and the same distance to the vessel wall, but different azimuthal angles. (I) (M), (J) (N), (K) (O), (L) (P) are pairwise data generated from four simulations with the platelets starting at the same azimuthal angle and same distance to the vessel wall, but different *z*-coordinates (5 µm, 0 µm, -5 µm, -10 µm separately). Note: (J) (N) are duplicates of (B) (F).

Quantitative analysis of RBCs-influenced platelet-thrombus interactions was carried out for different initial positions of the platelet (Figure 6). Some general trends are: (i) the distance between the platelet and RBCs exhibits “wave-like” oscillations, with each peak corresponding to the passing of an RBC; (ii) the disk-shaped platelet spends most of its time in a parallel orientation and experiences a fast flipping motion every 20~25 µm; (iii) such flipping motion is prolonged when the initial position of the platelet has a larger azimuthal angle (*x*-coordinate deviation from 7.5 µm). The third trend could have important implications during the development of hemostasis, since the longer duration of perpendicular orientation extends the time window of platelet-thrombus hydrodynamic interaction at the side of the thrombus. Such extension enhances the probability of successful recruitment of following platelets onto the side of the thrombus, compensating for the reduced margination effect of RBCs at larger azimuthal angles (the platelet-RBCs distance increases with increasing azimuthal angle of the initial position of the platelet, Figure 6A~D). It was also found that the closest distance between the thrombus and platelet throughout simulations is concurrent with the shortest separation distance between the platelet and RBCs (Figure 6A).

## Discussion

This study evaluates the hydrodynamic interaction between deformable RBCs, ellipsoidal platelets and a model thrombus shape under physiological flow inside a cylindrical arteriole. The flow profile inside the vessel was found to have stagnation zones directly upstream and downstream of the thrombus at most azimuth angles. In previous studies, stagnation zones were identified by *in vivo* experiments [36]. A new mechanism of platelet aggregation has been described by Nesbitt et al. and is considered to be mainly shear stress gradient dependent [40]. In their study, the authors found that platelets form aggregates when encountering a drop in shear rate. However, they did not explore the fact that platelets also experience a drop in shear rate at the opposite side of the thrombus and no aggregate is formed. Based on the present results, we argue that the non-axisymmetric placement of a bump-shaped thrombus creates a hydrodynamic environment which causes the magnitude of shear-gradient that platelets experience to differ by 3~4 fold at different azimuth positions (Figure 2). In other words, platelets experience a much smaller shear gradient at the opposite side of the thrombus, potentially explaining this phenomenon.

While the majority of the available blood flow velocity data comes from large human arteries, there is a lack of published information for the full range of percentage stenosis in vessels of smaller sizes (arterioles, venules and capillaries). We choose human carotid artery stenosis data [37] for comparison in Figure 3, recognizing the blood rheology difference (e.g., Hct) between large arteries (~5 mm in diameter for carotid artery in human adults) and small arterioles (~40 µm). Any comparison is limited to examining qualitative trends, due to the difference in length scales between the clinical and computational systems. Another point to note is the conversion of the *in vivo* data from diameter stenosis provided by Grant et al. to area of stenosis. It is known that the thrombus does not develop uniformly along the peripheral of the vessel wall to form a perfect ring shape (which would translate to a quadratic relationship between diameter and area), and does not develop in a “flow-chamber” character (which would translate linearly between diameter and area). Thus, the relation between diameter stenosis and area stenosis must be super-linear but not exceed a power of two. Indeed, a recent study determined with CT angiography that the correlation of carotid stenosis diameter with cross-sectional areas is super-linear but did not exceed a square dependence [41]. In Figure 3B, we thus assume a power of 1.5 and introduce an additional adjustment factor of 0.63 to compare the *in vivo* patient sample with the present simulation results. The factor 0.63 may represent the result of blood rheology differences due to the two orders of magnitude length scale difference between carotid arteries and a 15 µm diameter arteriole.

Due to the complex *in vivo* vessel/thrombus geometry, as well as the larger vessel diameters (45~60 µm) of mouse cremaster muscle (15 µm in our simulation), the convergence of platelet trajectories fluctuates in individual *in vivo* experiments in Figure 4. However, it shows a similar trend with comparable ranges. The mechanics of deformable RBCs cause the flow inside the vessel tube to deviate from the typical characteristics of Stokes flow. This can be understood by simple reasoning: Stokes flow does not require time to develop and the disturbance will immediately cause steady flow changes throughout the entire volume; however, RBCs do require time to establish their equilibrium ‘cap-shaped’ geometry following the start of flow.

The irreversible motion depicted in Figure 4D is in accordance with the results of Figure 6 (A)(B)(I)~(K), where one finds that platelets approach the thrombus faster and leave the thrombus slower. This may reduce the probability of the thrombus to grow in the direction of flow, increasing the effective wound coverage and reducing the size of the thrombus to result in a reduced risk of a blood clot. This non-Stokesian behavior is introduced into the system by the geometric nonlinearity and mechanical properties of the RBCs, with the elasticity of the RBCs adding an additional time scale.

It is well understood that RBCs contribute to the rheology of blood by increasing the viscosity and causing margination of platelets and leukocytes. However, the hydrodynamic interaction between platelets and a thrombus in the presence of RBCs has not been previously studied in detail. From Figure 6, one may conclude that there are three major sources for potential platelet deposition onto the thrombus, i.e., when the platelet comes close enough to the thrombus surface for molecular adhesion such as that mediated by α_IIb_β_3_-vWF-α _IIb_β_3_ bond formation. The first source of such platelet deposition potential is the geometry of the thrombus itself which reduces the platelet-thrombus distance in the thrombus region (*z* coordinates ranging from 15 to 30 µm) in all simulations performed. The second source is the platelet flipping motion, where the platelet rotational angle jumps (from π/2 to -π/2) are always concomitant with a dip in the platelet-thrombus distance (Figure 6), indicating the physiological importance of the ellipsoid shape of platelets. The third is the interaction between platelets and RBCs. These three sources of platelet deposition potential identified here not only reinforce the importance of their ellipsoid shape to normal platelet function, but also show the direct enhancement of platelet-thrombus interaction through platelet-RBCs interactions. In the future, it would be interesting to investigate the direct interaction between RBCs and the thrombus as well as any mechanical effect of RBCs on thrombus fragmentation. Previous studies show that RBCs can be engineered to suppress thrombosis by coupling them with anti-thrombotic agents [42], suggesting frequent physical contact between RBCs and the developing thrombus.

Ultrasound Doppler is currently the most common blood velocity measurement method and is widely accepted in clinical applications. Doctors judge patients’ degree of stenosis based on elevated blood flow velocity. While much faster, convenient and non-invasive compared to angiography, Doppler has its own limitation: accuracy. The major source of inaccuracy comes from the broad assumption that the direction of blood flow is parallel to the axis of the blood vessel lumen, which can cause miscalculation of the velocity magnitude [43]. Another limitation is that the Doppler data does not easily facilitate reconstruction of 3D flow fields and vessel conditions. Several improvements have been made to either precisely position the sample volume [44] or use multiple transducers to measure the velocity vector [45], but these improvements cannot fully eliminate uncertainty. Computational studies can better reveal the flow characteristics inside the complex geometry of the vessel wall thus helping clinicians to better link the Doppler data with patient symptoms. Comparisons such as that in Figure 3 serve as an example: from given velocity data, clinicians may assess the range of potential thrombus shape and position. This also suggests that simulation studies can potentially regain the lost dimension in clinical measurement (2D to 3D).

In conclusion, we have established a hydrodynamic model which is able to reveal the hydrodynamic interaction between blood cells and the vessel wall in 3D. We successfully characterized the hydrodynamic environment for different thrombus shapes and degree of stenosis and the results agree with *in vivo* results. Future applications of the present model will be focused on adhesion of various types of receptor-ligand pairs. We have already incorporated the kinetics of GPIbα–vWF-A1 into our Platelet Adhesion Dynamics model (PAD) [5]. We also successfully recover the translocational motion of platelets on injured vessel walls [24]. It will be interesting to examine how platelets tether and translocate under the interaction of surrounding RBCs and thrombus in a cylindrical vessel.
